# Effectiveness of testosterone therapy in obese men with low testosterone levels, for losing weight, controlling obesity complications, and preventing cardiovascular events: Protocol of a systematic review of randomized controlled trials

**DOI:** 10.1097/MD.0000000000010482

**Published:** 2018-04-27

**Authors:** Amanda S. Mangolim, Leonardo A. R. Brito, Vania S. Nunes-Nogueira

**Affiliations:** Department of Internal Medicine, São Paulo State University (UNESP), Medical School, Botucatu, São Paulo, Brazil.

**Keywords:** low testosterone, metabolic syndrome, obesity, testosterone therapy, type 2 diabetes mellitus, weight loss

## Abstract

**Background:**

The use of testosterone replacement therapy in obese men with low testosterone levels has been controversial. This review aims to analyze the effectiveness of testosterone therapy for weight loss and preventing cardiovascular complications in obese men with low testosterone levels.

**Methods:**

We will perform a systematic review according to Cochrane Methodology of randomized studies, including crossover studies, wherein patients are allocated into one of the two groups: testosterone therapy and control (no treatment or placebo). The primary outcomes analyzed will be: weight loss, adverse events, quality of life, improvement of libido, control of obesity complications, frequency of cardiovascular events, and deaths. Four general and adaptive search strategies have been created for the following electronic health databases: Embase, Medline, LILACS, and CENTRAL. Two reviewers will independently select the eligible studies, assess the risk of bias, and extract the data from included studies. Similar outcomes measured in at least two trials will be plotted in the meta-analysis using Review Manager 5.3. The quality of evidence of the effect estimate of the intervention for the outcomes that could be plotted in the meta-analysis will be generated according to the Grading of Recommendations Assessment, Development, and Evaluation (GRADE) Working Group.

**Results:**

Although testosterone replacement seems to be an attractive treatment modality for obese men with low testosterone, its potential benefits has been refuted by some studies, whose results have not shown significant differences between treated and untreated patients.

**Conclusion:**

For obese men with low testosterone concentrations, the proposed systematic review aims to answer the following questions: When compared with no treatment or placebo: Is testosterone therapy safe? Is testosterone therapy effective in promoting weight loss, a sustained reduction in body weight and changes in body composition? Is testosterone effective in improving quality of life, libido, and erectile function? Is testosterone therapy effective in controlling obesity complications and in preventing cardiovascular events?

## Abstract 2

Supplemental Digital Content is available in the text

## 1 Introduction

### 1.1 Description of the condition

Obesity is a complex chronic condition with serious social and psychological dimensions that affects all ages and different socioeconomic groups in all countries.^[1]^ According to the World Health Organization, obesity affects about 400 million adults globally, with an alarming increase in its prevalence in recent years.^[2]^ It is estimated that the worldwide prevalence of obesity was 9.8% in men in 2008, which was almost twice that in the 1980s.^[1]^

It is known that excess body weight especially central obesity is an independent risk factor for increased morbidity and mortality, not only for cardiovascular causes, but also for type 2 diabetes mellitus (type 2 DM), cancer, and musculoskeletal disorders, and complications cause around 3 million deaths per year globally.^[3]^ Excess body fat is considered responsible for most risk factors associated with obesity and increased mortality, independent of body mass index (BMI).^[3]^ Despite this knowledge, obesity has been a major public health problem and one of the most neglected diseases.^[1]^

Several studies have shown that, in individuals with obesity or overweight, intensive lifestyle changes, which include nutritional counseling and physical activity, are able to reduce body weight and insulin resistance, preventing the progression of type 2 DM and cardiovascular diseases.^[1]^ Nevertheless, it is known that adequate and sustained weight loss is very difficult to achieve.^[3]^ Because of this, many researchers have studied different ways to promote effective weight loss.

It is known that obesity in men is associated with low testosterone levels and there is an inverse relationship between abdominal circumference and serum testosterone concentrations.^[4,5]^ This association is extremely relevant; some authors have pointed out that obesity is one of the most important risk factors for testosterone level reduction, and more important than age and other chronic diseases.^[3]^

Obese men have 30% lower total testosterone levels than eutrophic men and 40% have levels below the lower limit of normality.^[3]^ Although the exact pathogenic mechanisms are not yet clarified, a decline in sex hormone-binding globulin associated with obesity may partially explain the observed decrease in testosterone concentrations.^[6]^

In addition, it is believed that adipokines and proinflammatory mediators originated in adipose tissue may also play a role in suppression of the hypothalamic-pituitary-gonadal axis.^[7]^ In patients with severe obesity, besides the low total testosterone levels, free and bioavailable testosterone concentration also decreases, and this seems to be caused by this axis suppression.^[8]^

Male obesity is also associated with increased aromatase activity within adipocytes resulting in peripheral conversion of testosterone into estradiol and subsequent increase in estradiol levels. In turn, estradiol exerts a negative feedback effect on LH secretion, reducing the plasma concentrations of testosterone.^[9]^

Actually, this inverse relationship of testosterone with visceral adipose tissue is bidirectional. Studies in men with androgen deprivation therapy due to prostate cancer have shown that low testosterone leads to “sarcopenic obesity” associated with insulin resistance^[10]^; conversely, uncontrolled studies have evidenced that testosterone therapy reduces fat and increases muscle mass,^[11]^ and also waist circumference (WC) has been invariably reduced by testosterone treatment in controlled studies.^[12]^

Biochemical deficiency of androgens in obese men may be partially reversed with weight loss^[13]^ and, as a form of treatment for these individuals, it is recommended to modify the lifestyle to lose weight.^[4]^

Many randomized clinical trials have evaluated the impact of diet and physical activity on testosterone levels in obese men. The results of these studies are essentially conflicting. Some showed increased testosterone,^[14–16]^ others showed no change,^[17,18]^ and a small study showed a decrease in testosterone levels.^[19]^

Corona et al^[1]^ performed a systematic review on body weight loss and hypogonadotropic hypogonadism. They included observational and interventional studies, and weight loss by either bariatric surgery or low-calorie diet resulted in a significant increase in gonadotropins and testosterone (total and free) levels, and multiple regression analysis demonstrated that the degree of body weight loss was the best determinant of total testosterone rise.

Therefore, body weight loss and lifestyle interventions should be the first approach offered to obese men with low testosterone levels.

However, considering the difficulty in achieving weight loss even with changes in lifestyle, and the possibility of regaining weight in the long term, we asked the following question: in obese men with low testosterone levels and with or without metabolic syndrome or type 2 DM, what is the effectiveness of testosterone therapy for weight loss and improvement of cardiovascular complications compared with no replacement?

### 1.2 Description of intervention

Testosterone therapy has been recommended for symptomatic men with classical hypogonadism diagnosis, which includes men with primary testicular failure, central defects of the hypothalamus or pituitary cause, or dual defects that affect both the testis and pituitary gland. In these cases, the therapy aims to induce and maintain secondary sex characteristics and improve sexual function, sense of well-being, and bone mineral density.^[20]^

There are many forms of testosterone therapy, and intramuscular injection applied every 2 to 4 weeks is one of the most common and inexpensive modalities.^[20]^ These generally contain 1 or more testosterone esters; their principal forms are testosterone cypionate and testosterone enanthate. Testosterone undecanoate is a relatively recent injectable long-actioning testosterone, and, in countries where it is commercialized, the recommended dose is intramuscular 1000 mg, followed by 1000 mg at week 6, and 1000 mg every 10 to 14 weeks.^[20]^

Testosterone replacement can also be administered via the buccal mucosa and transdermally using patches, gels, or liquids. Transdermal forms of therapy seem to provide an important therapeutic benefit; however, these gels, ointments, or patches must be administered daily and the adherence to them can be improper.^[21]^ The following are examples of these formulations: 1% and 2% testosterone gel, transdermal testosterone patch, buccal bio adhesive testosterone tablets, and testosterone-in adhesive matrix patch.^[20]^ The most recommended regimes are one or two 5-mg testosterone patches applied nightly over the skin of the back or upper arm, 5 to 10 g of testosterone gel applied daily over a covered area of skin, 30 mg of a bio-adhesive, buccal testosterone tablet applied to the buccal mucosa twice daily; in testosterone pellets, the dose and regimen are according to the formulation used.^[20]^

### 1.3 Adverse events of the intervention

Studies in young, hypogonadal men have found a low frequency of adverse events with the replacement of testosterone. Common adverse events for which there is evidence of association with testosterone therapy include erythrocytosis, acne, oiliness of skin, increase in prostate-specific antigen (PSA) and prostate volume, growth of metastatic prostate cancer, and reduced sperm production and fertility.^[20]^ Uncommon adverse events for which there is weak evidence of association with testosterone administration are gynecomastia, male pattern baldness (familial), and growth of breast cancer.^[20]^

Some adverse effects are formulation-specific. For example, short-acting intramuscular forms have been associated with fluctuation in mood or libido, pain at the injection site, excessive erythrocytosis, and coughing episodes immediately after the injection with testosterone undecanoate long-acting. Transdermal gel and patches have been associated with skin irritation, and the former presents a potential risk for testosterone transfer to partner or anyone who is in close contact. Buccal testosterone tablets can cause alterations in taste, and irritation of gums, and pellet implants can cause local infection.^[20]^

### 1.4 How the intervention might work

Besides classical hypogonadism causes, testosterone has been also prescribed for other health-related conditions, as in obese men with symptomatic testosterone deficiency.

In men with obesity and severe obstructive sleep apnea, testosterone replacement increases muscle mass and decreases fat mass independently of low or normal testosterone levels.^[13]^ In patients with low testosterone and type 2 DM, Magnussen showed that testosterone therapy decreased visceral abdominal fat and improved insulin sensitivity.^[22]^ Kapoor and collaborates in a crossover study evaluated testosterone therapy in obese men with type 2 DM and low testosterone.^[23]^ Although no significant changes were observed in BMI, there was a significant reduction in WC following testosterone replacement. In a study by Kalinchenko and collaborators,^[24]^ 184 obese men with metabolic syndrome and total testosterone levels <12 nmol/L were randomized to receive for 30 weeks either parenteral testosterone undecanoate or placebo. During the follow-up, there were significant decreases in weight, BMI, and WC in the intervention group compared with those in the placebo group. In a study by Saad and collaborates,^[25]^ long-term testosterone therapy in obese hypogonadal men resulted in substantial and sustained reductions in body weight, WC, and BMI.

From these results, some physicians have recommended testosterone replacement as a novel and useful therapeutic strategy for the treatment and management of obesity.^[12]^

The American Association of Clinical Endocrinologists and American College of Endocrinology recommend men with hypogonadism (defined as symptoms associated with low testosterone) and obesity who are not seeking fertility should be considered for testosterone therapy in addition to lifestyle intervention since testosterone in these patients results in weight loss, decreased WC, and improvements in metabolic parameters.^[4]^

One explication for the improvement of metabolic control and weight loss in testosterone therapy is that this hormone increases lean body mass, thus increasing resting energy expenditure.^[12]^ In addition, androgens regulate body composition by promoting the commitment of mesenchymal pluripotent cells into myogenic lineage and inhibiting their differentiation into the adipogenic line.^[26]^ Testosterone regulates carbohydrates, proteins, and fat metabolism,^[1,27]^ and testosterone therapy in men with testosterone deficiency results in the normalization of glucose utilization and increased lipid oxidation.^[28]^ Further, testosterone therapy improves erectile function and increases vigor and reduces fatigue,^[12]^ promoting with this a better disposition to perform exercises.

### 1.5 Why it is important to perform this review?

Although testosterone replacement seems to be an attractive treatment modality for obese men with low testosterone, its potential benefits for weight loss, improvement of sexual function, and obesity-related morbidity have been refuted by some studies, whose results have not shown significant differences between treated and untreated patients.

Ng Tang Fui and collaborates^[6]^ randomized 100 obese men (BMI > 30) with testosterone levels ≤12 nmol/L to either a testosterone therapy or placebo group, and the study intervention group showed significant improvement in Aging Male Symptoms Scale (AMS) score and erectile function; however, the final weight was the same between the groups.

Cai and collaborates,^[29]^ in a systematic review published in 2014, evaluated the metabolic effects of testosterone replacement in hypogonadal men with type 2 DM. The meta-analysis of included studies showed a significant difference in favor of the intervention for fasting plasma glucose (5 studies) and HbA1c reduction (3 studies); however, there was no statistically significant difference in final body fat in the 3 studies that analyzed this outcome. The authors did not include studies with obese individuals without either type 2 DM or metabolic syndrome.

In another recently published systematic review that studied the efficacy of testosterone replacement in men with hypogonadism, testosterone significantly improved the scores of AMS and increased lean body mass; however, no significant differences were identified in BMI and reduction of fat mass in the 5 randomized studies that evaluated these outcomes.^[30]^ It is important to emphasize that this review excluded studies with diabetic patients.

In conclusion, the use of testosterone in obese men has been controversial in terms of weight loss and control of obesity-related diseases (metabolic syndrome, type 2 DM, and others cardiovascular disorders). Compared with no replacement, some studies demonstrated benefits and others did not show evidence of significant differences. In addition, the systematic reviews published on this subject have not included obese men with or without related complications as a patient eligibility criterion^[29,31,32]^; conversely, some excluded these individuals.^[30]^

## 2 Objectives

This review aims to analyze if obese men with low testosterone levels following testosterone replacement show evidence for weight loss and sustained reductions in body weight, decreasing body fat, gaining muscle mass, improving quality of life, libido, and erectile function, controlling obesity complications (type 2 DM, hypertension, dyslipidemia, obstructive sleep apnea, depression), and preventing cardiovascular events and deaths.

For obese men with low testosterone concentrations with or without metabolic syndrome or type 2 DM, the proposed systematic review aimed to answer the following questions:

1. When compared with no treatment or placebo, is testosterone replacement therapy effective in promoting weight loss?
2. When compared with no treatment or placebo, is testosterone replacement therapy effective in promoting a sustained reduction in body weight?
3. When compared with no treatment or placebo, is testosterone replacement therapy effective in promoting changes in body composition (increases in lean body mass and decreases in total fat mass)?
4. When compared with no treatment or placebo, is testosterone replacement therapy effective in promoting the improvement in quality of life, libido, and erectile function?
5. When compared with no treatment or placebo, is testosterone replacement therapy effective in controlling obesity complications?
6. When compared with no treatment or placebo, is testosterone replacement therapy effective in preventing cardiovascular events and deaths?
7. Is testosterone replacement therapy safe?

## 3 Methods and analyses

This systematic review will be conducted according to the Cochrane Collaboration^[33]^ and reported according to the PRISMA Statement.^[34]^ This protocol contains the 17 items considered to be essential in a systematic review according to PRISMA-P.^[35]^

### 3.1 Eligibility criteria

#### 3.1.1 Types of studies

We will include randomized studies and crossover studies, in which the patients are allocated into 1 of the 2 groups: testosterone therapy or control (no treatment or placebo).

#### 3.1.2 Participants

Obese men older than 18 years with low testosterone levels.

### 3.2 Diagnostic criteria

We will consider men with BMI ≥ 30 kg/m^2^ as well as those with BMI < 30 kg/m^2^ but presenting with central obesity (WC higher than ethnic specific values), as obese.^[36]^ We included patients who may or may not have metabolic syndrome, type 2 DM, obstructive sleep apnea, as well as other consequences of obesity. The eligible randomized studies that included patients with one of these complications did not specify any obesity criteria; however, we will include those whose BMI or WC at baseline in both the intervention and control groups were according to the aforementioned criteria.

The range of reference values of total testosterone may vary in different laboratories. In some, the lower limit level in healthy young men is 280 to 300 ng/dL (9.8–10.4 nmol/L).^[20]^ The same occurs with the lower limit of the normal range for serum-free testosterone level; some reference laboratories use 5 to 9 pg/mL (0.17–0.31 nmol/L) as the normal range.^[20]^ Thus, the Endocrine Society's recommendation is that clinicians should use the lower limit of normal range for the definition of low testosterone for health young men established in their laboratory.

The International Society for the Study of the Aging Male recommends 12.1 nmol/L (350 ng/dL) as a lower limit of normality for total testosterone, but testosterone replacement therapy may be reasonably considered with testosterone levels higher than 12.1 nmol/L, based on symptoms and if free testosterones are reduced.^[37]^

According to the European Association of Urology, both immunoassay- and mass spectrometry-based assays can produce reliable results for testosterone concentrations, and evaluation should be based on reference ranges for normal men provided by the laboratory measuring the samples.^[38]^

In this review, we will consider the following as low testosterone: total testosterone concentrations ≤350 ng/dL. Values higher than this (either for total or free testosterone) will be considered if indicated by the authors as the lower limit of the normal range for healthy young men in their laboratory.

As several obese men may not present with the typical signs and symptoms related to hypogonadism, especially in the initial stages,^[9]^ we will include studies with symptomatic and asymptomatic patients.

#### 3.2.1 Types of interventions

The intervention group will be composed of patients undergoing testosterone replacement therapy for at least 8 weeks, which may be administered in an injectable, transdermal, gel, topical, or adhesive form.

These individuals should also be advised to change their lifestyle and receive standard treatment for obesity-related comorbidities.

#### 3.2.2 Comparison

The comparison group will receive either placebo or nontestosterone replacement therapy associated with lifestyle recommendations and standard treatment of diseases related to obesity.

### 3.3 Exclusion criteria

We will exclude studies wherein the population was predominantly composed of men with contraindications to the use of testosterone, that is, men with breast cancer, prostate cancer, palpable nodule or induration in prostate, PSA > 4 or >3 ng/mL in men at high risk for prostate cancer, hematocrit >50%, severe sleep apnea obstructive syndrome that did not respond to the recommended treatment, decompensated heart failure and men with a diagnosis of established classical central or peripheral hypogonadism (hypopituitarism; hyperprolactinemia; gonadal dysgenesis; testicular neoplasms; and Klinefelter, Noonan, Prader Willi, and other syndromes).

#### 3.3.1 Types of outcome measures

##### 3.3.1.1 Primary outcomes

The primary outcomes analyzed will be weight loss (measured by final weight or WC or BMI), adverse events (frequency of erythrocytosis, acne, oiliness of skin, prostate neoplasia, gynecomastia, male pattern baldness, growth of breast cancer, induction or worsening of obstructive sleep apnea, side effects formulation specifics), quality of life (that can be evaluated by AMS scale or other questionnaire), control of obesity complications (type 2 DM, hypertension, dyslipidemia, obstructive sleep apnea, depression), and frequency of cardiovascular events and deaths.

##### 3.3.1.2 Secondary outcomes

The secondary outcomes will be increase of lean body mass and decrease of total fat mass, improvement of symptoms of hypogonadism (improvement of sexual desire and erectile function), sustained weight loss, and normalization of testosterone level, and increase of PSA levels.

#### 3.3.2 Timing

Included studies should have a follow-up duration of at least 8 weeks.

### 3.4 Search methods for identification of studies

#### 3.4.1 Electronic database

Four general and adaptive search strategies have been created for the following electronic health databases: Embase (by Elsevier, 1980–2018), Medline (by PubMed, 1966–2018), LILACS (by Virtual Health Library, 1982–2018), and the Registry of Controlled Clinical Studies of the Cochrane Collaboration (CENTRAL—Cochrane). The mesh terms and synonyms of obesity, metabolic syndrome, type 2 DM, and testosterone will be used. There will be no language or year restriction. A draft Medline search strategy is included in Appendix 1.

We will use the Endnote software to download all references in order to remove duplicates and facilitate the selection process.

#### 3.4.2 Searching other resources

The following databases will also be searched for eligible studies: Trip database, SCOPUS, Web of Science, CINAHL, Australasian Medical Index, and Chinese Biomedical Literature Database. We will also look for unpublished studies on the ClinicalTrials.gov website, Brazilian Registry of Clinical Trials (Rebec), and the gray literature, through abstracts published in annals and lectures of congress.

In primary or secondary relevant studies identified, we will check for more eligible studies in their cited/included articles.

### 3.5 Data collection and analysis

#### 3.5.1 Selection of studies

Two reviewers will independently select the titles and abstracts identified by the bibliographic research. The studies potentially eligible for inclusion in the review will be selected for full reading and subsequently assessed for adequacy to the proposed participants/intervention/comparison/outcomes (PICO). In case of disagreements, there will be a debate between the reviewers and a third party (VSNN) before the final decision.

**Figure 1:**
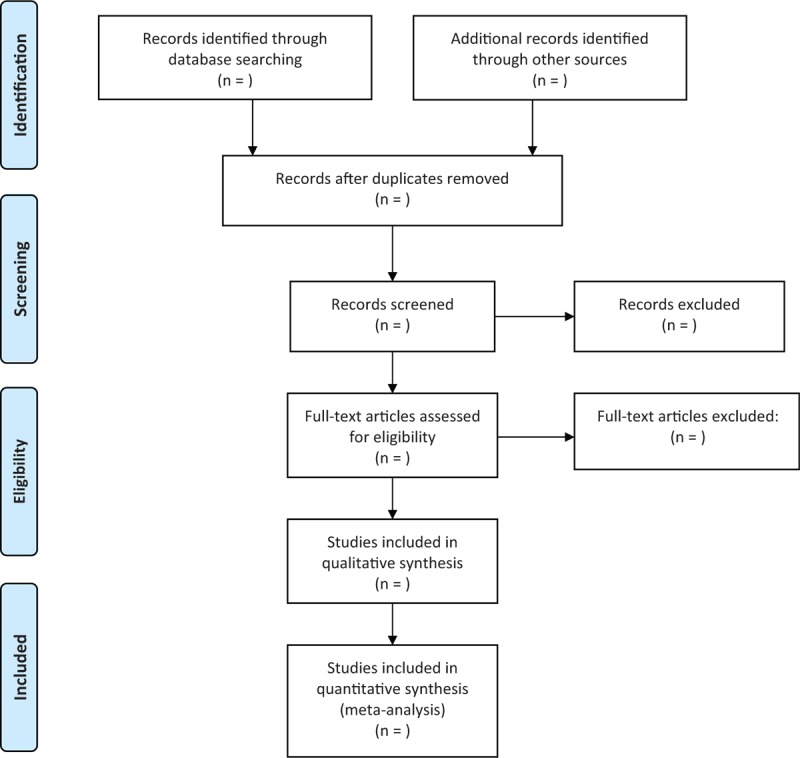
Flow diagram of selected studies.

The study selection flowchart will be created according to the PRISMA guidelines (Fig. 1).

### 3.6 Data extraction and management

Both reviewers will use an extraction form for the selected studies in order to register the year of publication, trial size, duration of follow-up, information pertaining to the eligibility criteria (inclusion and exclusion criteria), name, dosage, frequency and administration of intervention, type of control (placebo or no treatment), and all outcomes. We also will extract patient baseline characteristics (mean age, weight, BMI and WC, associated diseases, mean testosterone concentrations), and the methodology quality of each study.

To ensure consistency between reviewers, we will conduct a calibration exercise before starting the review.

In case of duplicate publications or more reports from the primary study, data extraction will be optimized using the best available information for all items from the same study.

There will be a debate between the reviewers and VSNN in case of disagreement between the reviewers in this process.

### 3.7 Assessment of bias in included studies

For each clinical experiment selected, the risk of bias will be evaluated according to the criteria described in the Cochrane Handbook for Systematic Reviews of Interventions,^[33]^ which considers 7 domains: random sequence generation, allocation concealment, blinding of participants and personnel, blinding of outcome assessment, incomplete outcome data, selective reporting, and other bias. Each of the other items will be sorted by the 2 reviewers (ASM and LARB) as having low risk of bias, high risk of bias, or unclear. In case of disagreements, there will be a debate between the reviewers and VSNN before the final classification.

### 3.8 Measure of treatment effect

For dichotomous data, the relative risk will be calculated with the 95% confidence interval (CI) as the estimate of effect of the intervention. Continuous data will be expressed as means and standard deviation, and the difference between the means with 95% CI. If possible, the continuous data will be transformed into dichotomous data (e.g., rate of patients that achieved WC < 94 cm).

### 3.9 Unit of analysis issues

The unit of analysis will be the individual participants. In case of crossover studies, only data from the first phase will be considered.

### 3.10 Dealing with missing data

The authors of the original studies will be contacted, if necessary, to obtain missing information for each included study. We will use only the available data (in the published articles or provided by their contact authors). We will not use any method to input the missing data. If available, we preferentially will use data from intention-to-treat analysis.

### 3.11 Assessment of heterogeneity

The inconsistency between the results of the included studies will be ascertained by the visual inspection of the forest plot (no overlap of CIs around the effect estimates of the individual studies) and by the Higgins or I^2^ test, in which I^2^ > 50% indicates a moderate probability of heterogeneity. The causes of potential heterogeneity between the studies will also be planned and evaluated.

### 3.12 Assessment of reporting biases

For a specific outcome, if more than 10 studies are included in the meta-analysis, we will use the funnel plot and Egger regression test to investigate the presence of reporting bias.^[39]^

### 3.13 Data synthesis

Similar outcomes measured in at least 2 trials will be plotted in the meta-analysis using Review Manager 5.3 (Review Manager [RevMan], version 5.3; Copenhagen: The Nordic Cochrane Center, The Cochrane Collaboration, 2014). We will select the random effects as the analysis model in the meta-analysis. The method of inverse variance and Mantel–Haenszel will be the statistical methods used to establish the effect estimates between the continuous and dichotomy data, respectively, of the studies included.

### 3.14 Subgroup analysis

If enough data are available, we plan to perform subgroup analysis according to presence or absence of hypogonadal symptoms and according to the following diagnosis: metabolic syndrome, type 2 DM, and obesity without these 2 complications. We also plan to analyze the outcomes up to 6 months, between 6 and 12 months, more than 1 year after the start of treatment, where will we consider short-, medium-, and long-term follow-ups, respectively. Other subgroup analysis will be related to the type of testosterone replacement (injection, gel, patch, brand, etc.).

### 3.15 Sensitivity analysis

If possible, we plan to perform sensitivity analysis restricting it to studies with low risk of selection, detection, and attrition bias.

### 3.16 Grading the quality of evidence

The quality of evidence of the effect estimate of the intervention for the outcomes that could be plotted in the meta-analysis will be generated according to the Grading of Recommendations Assessment, Development, and Evaluation (GRADE) Working Group.^[40]^

The GRADE evaluates the quality of the totality of the evidence of certain technology in health on an outcome, specifically, the most important outcomes from the patient's perspective. Randomized studies have the best quality of evidence, but the quality deteriorates if the studies have great limitations that may interfere with the treatment effects’ estimates. These limitations include the risk of the bias mentioned above, inconsistencies, indirect evidence, imprecision, and publication bias of the results of every outcome analyzed.

### 3.17 Ethics dissemination

As no primary data collection will be undertaken, no formal ethical assessment is required by our institution. We plan to present the findings of this systematic review in a peer-reviewed scientific journal. We also intend to present it, including preliminary findings, at the appropriate conferences.

### 3.18 Amendments

Regarding our protocol registered on Prospective register of Systematic Reviews, we have performed a few modifications as follows. We extended the title to make it more informative, reflecting the PICO approach. For the same reason, we enlarged our review question. As justified above regarding patient eligibility criteria, we modified the low testosterone diagnosis of <300 ng/dL (10.4 nmol/L) to ≤350 ng/dL (12.1 nmol/L), with the possibility to accept values higher than this if indicated by the authors as the lower limit of normal range for healthy young men. We expanded the diagnosis criterion of central obesity (WC higher than specific values for ethnicity).

## Acknowledgments

The authors thank Dr. Lehana Thabane to motivate us to publish this systematic review protocol and the authors thank Editage to review the language of our manuscript.

## Author contributions

**Conceptualization:** Amanda S. Mangolim, Leonardo A. R. Brito, Vania dos Santos Nunes-Nogueira.

**Formal analysis:** Amanda S. Mangolim.

**Funding acquisition:** Leonardo A. R. Brito, Vania dos Santos Nunes-Nogueira.

**Investigation:** Amanda S. Mangolim, Leonardo A. R. Brito.

**Methodology:** Amanda S. Mangolim, Vania dos Santos Nunes-Nogueira.

**Project administration:** Vania dos Santos Nunes-Nogueira.

**Resources:** Leonardo A. R. Brito, Vania dos Santos Nunes-Nogueira.

**Supervision:** Vania dos Santos Nunes-Nogueira.

**Validation:** Leonardo A. R. Brito.

**Writing – original draft:** Amanda S. Mangolim, Vania dos Santos Nunes-Nogueira.

**Writing – review and editing:** Vania dos Santos Nunes-Nogueira.

## Supplementary Material

Supplemental Digital Content
