# Supplementary material for: Effectiveness of testosterone therapy in obese men with low testosterone levels, for losing weight, controlling obesity complications, and preventing cardiovascular events: Protocol of a systematic review of randomized controlled trials
Source: Medicine (Baltimore). 2018 Apr 27;97(17):e0482. doi: 10.1097/MD.0000000000010482 (PMC5944503; doi:10.1097/MD.0000000000010482)
Supplement: Supplemental Digital Content [file medi-97-e0482-s001.docx]

**APPENDIX 1**

**Draft Medline search – PubMed interface**

#1 "Obesity"[Mesh] OR "Obesity, Abdominal"[Mesh] OR (Abdominal Obesities) OR (Abdominal Obesity) OR (Obesities, Abdominal) OR (Central Obesity) OR (Central Obesities) OR (Obesities, Central) OR (Obesity, Central) OR (Obesity, Visceral) OR (Obesities, Visceral) OR (Visceral Obesities) OR (Visceral Obesity) OR "Obesity, Morbid"[Mesh] OR (Morbid Obesities) OR (Obesities, Morbid) OR (Obesity, Severe) OR (Obesities, Severe) OR (Severe Obesities) OR (Severe Obesity) OR (Morbid Obesity) OR "Obesity, Metabolically Benign"[Mesh] OR (Benign Obesity, Metabolically) OR (Metabolically Healthy Obesity) OR (Healthy Obesity, Metabolically) OR (Metabolically Benign Obesity) OR "Abdominal obesity metabolic syndrome" [Supplementary Concept] OR (Abdominal Obesity-Metabolic Syndrome) OR "Diabetes Mellitus, Type 2"[Mesh] OR (Diabetes Mellitus, Noninsulin-Dependent) OR (Diabetes Mellitus, Ketosis Resistant) OR (Ketosis-Resistant Diabetes Mellitus) OR (Diabetes Mellitus, Non Insulin Dependent) OR (Diabetes Mellitus, Non-Insulin-Dependent) OR (Non-Insulin-Dependent Diabetes Mellitus) OR (Diabetes Mellitus, Stable) OR (Stable Diabetes Mellitus) OR (Diabetes Mellitus, Type II) OR(NIDDM) OR (Diabetes Mellitus, Noninsulin Dependent) OR (Diabetes Mellitus, Maturity-Onset) OR (Diabetes Mellitus, Maturity Onset) OR (Maturity-Onset Diabetes Mellitus) OR (Maturity Onset Diabetes Mellitus) OR (MODY) OR (Diabetes Mellitus, Slow-Onset) OR (Diabetes Mellitus, Slow Onset) OR (Slow-Onset Diabetes Mellitus) OR (Type 2 Diabetes Mellitus) OR (Noninsulin-Dependent Diabetes Mellitus) OR (Noninsulin Dependent Diabetes Mellitus) OR (Maturity-Onset Diabetes) OR (Diabetes, Maturity-Onset) OR (Maturity Onset Diabetes) OR (Type 2 Diabetes) OR (Diabetes, Type 2) OR (Diabetes Mellitus, Adult-Onset) OR (Adult-Onset Diabetes Mellitus) OR (Diabetes Mellitus, Adult Onset) OR "Metabolic Syndrome X"[Mesh] OR (Insulin Resistance Syndrome X) OR (Syndrome X, Metabolic) OR (Syndrome X, Insulin Resistance) OR (Metabolic X Syndrome) OR (Syndrome, Metabolic X) OR (X Syndrome, Metabolic) OR (Dysmetabolic Syndrome X) OR (Syndrome X, Dysmetabolic) OR (Reaven Syndrome X) OR (Syndrome X, Reaven) OR (Metabolic Cardiovascular Syndrome) OR (Cardiovascular Syndrome, Metabolic) OR (Cardiovascular Syndromes, Metabolic) OR (Syndrome, Metabolic Cardiovascular) OR "Abdominal obesity metabolic syndrome" [Supplementary Concept] OR (Abdominal Obesity-Metabolic Syndrome)

#2 "Testosterone"[Mesh] OR (17-beta-Hydroxy-4-Androsten-3-one)OR (17 beta Hydroxy 4 Androsten 3 one) OR (Auxilium Pharmaceuticals Inc. Brand of Testosterone) OR (Testim) OR (GlaxoSmithKline Brand of Testosterone) OR (SmithKline Beecham Brand of Testosterone) OR (Andropatch) OR (17 beta Hydroxy 8 alpha 4 Androsten 3 one) OR (17-beta-Hydroxy-8 alpha-4-Androsten-3-one) OR (8 Isotestosterone) OR (8-Isotestosterone) OR (Schering Brand of Testosterone) OR (Unimed Brand of Testosterone) OR (Solvay Brand of Testosterone) OR (AndroGel) OR (Testosterone Sulfate) OR (Bartor Brand of Testosterone) OR (Testopel) OR (Pasadena Brand of Testosterone) OR (Testolin) OR (Ortho Brand of Testosterone) OR (Ferring Brand of Testosterone) OR (Testoderm) OR (CEPA Brand of Testosterone) OR (AstraZeneca Brand of Testosterone) OR (Paladin Brand of Testosterone) OR (Faulding Brand of Testosterone) OR (Watson Brand of Testosterone) OR (Androderm) OR (Sustanon) OR (Ulmer Brand of Testosterone) OR (Sterotate) OR (Hauck Brand of Testosterone) OR (Histerone) OR (Dr. Kade Brand of Testosterone) OR (Androtop) OR "Testosterone Congeners"[Mesh] OR (Androgens, Synthetic) OR (Androgen Analogs) OR (Analogs, Androgen) OR (Androgen Analogues) OR (Analogues, Androgen) OR (Anabolic Steroids) OR (Steroids, Anabolic) OR (Synthetic Androgens) OR "Testosterone Propionate"[Mesh] OR (TestosteronpropionatEifelfango) OR (propionatEifelfango, Testosteron) OR (Eifelfango Brand of Testosterone Propionate) OR (Virormone) OR (Ferring Brand of Testosterone Propionate) OR (Agovirin) OR "testosterone enanthate" [Supplementary Concept] OR (testosterone heptanoate) OR (testosterone heptylate) OR (Delatestryl) OR (Theramed brand of testosterone enanthate) OR (BTG brand of testosterone enanthate) OR (Durathate) OR (Roberts brand of testosterone enanthate) OR (Theramex) OR (Testosteron Depot-Rotexmedica) OR (Rotexmedica brand of testosterone enanthate) OR (Testosteron-Depot Eifelfango) OR (Eifelfango brand of testosterone enanthate) OR (Testosteron-Depot Jenapharm) OR (Jenapharm brand of testosterone enanthate) OR (Testrin P.A.) OR (Pasadena brand of testosterone enanthate) OR (Andropository) OR (Rugby brand of testosterone enanthate) OR (Primoteston Depot) OR (Schering brand of testosterone enanthate) OR "testosterone 17 beta-cypionate" [Supplementary Concept] OR (testosterone cypionate) OR (testosterone 17 beta-cyclopentanepropionate) OR (testosterone 17 beta-cyclopentylpropionate) OR (Depo-Testosterone) OR (Depo-Testosterone Cypionate) OR (deposteron) OR (Duratest) OR (Testa-C) OR (TestexElmu) OR (Andronate) OR (Depostomead) OR "testosterone-17-succinate" [Supplementary Concept] OR (testosterone hydrogen succinate) OR (testosterone-17-hemisuccinate) OR (T-17-HS) OR (testosterone hemisuccinate) OR (testosterone-17-succinate, sodium salt, (17beta)-isomer) OR "testosterone-17-sulfate" [Supplementary Concept] OR (testosterone 17-sulphate) OR (testosterone-17-sulfate, sodium salt) OR (testosterone-17-sulfate, ammonium salt) OR (testosterone-17-sulfate, (17alpha)-isomer) OR "testosterone glucuronate" [Supplementary Concept] OR ((alpha)-isomer of testosterone glucuronate) OR (epitestosteroneglucuronide) OR (testosterone 17-glucosiduronate) OR (testosterone glucuronide) OR "testosterone undecanoate" [Supplementary Concept] OR (testosterone undecylate) OR (Undestor) OR (Andriol) OR (Restandol) OR (Organon brand of testosterone undecanoate) OR (Pantestone) OR (Nebido) OR "Epitestosterone"[Mesh] OR (17-alpha-Testosterone) OR (17 alpha Testosterone) OR "testosterone 3-(O-carboxymethyl)oxime" [Supplementary Concept] OR (testosterone 3-CMOx) OR "testosterone-19-hemisuccinate" [Supplementary Concept] OR (testosterone-19-succinate) OR (t-19-h) OR "testosterone 17-phenylpropionate" [Supplementary Concept] OR (testosterone phenylpropionate) OR (Retandrol) OR "dimeric testosterone" [Supplementary Concept] OR (testosterone succinate dimerbis(3-oxo-4-estren-17 beta-yl) succinate) OR "9-fluoro-11-hydroxybenzo(d,e)testosterone 17-acetate" [Supplementary Concept] OR (9 alpha-fluoro-11 beta-hydroxybenzo(d,e)testosterone 17-acetate) OR "testosterone 17beta-N,N-dimethylglycinate hydrochloride" [Supplementary Concept] OR (TSDG cpd) OR "testosterone oxime" [Supplementary Concept] OR (17-hydroxyandrost-4-en-3-one oxime) OR (3-oximinoandrost-4-en-17-ol) OR "testosterone cyclodextrin" [Supplementary Concept] OR "testosterone isocaproate" [Supplementary Concept] OR "4-(carboxymethylmercapto)testosterone" [Supplementary Concept] OR (4-CMMT) OR "testosterone-3-carboxymethyloxime" [Supplementary Concept] OR (T-3-CMO) OR "testosterone palmitate" [Supplementary Concept] OR "testosterone 17-cyclohexanecarboxylate" [Supplementary Concept] OR (Benzotest) OR "testosterone decanoate" [Supplementary Concept] OR "testosterone acetate" [Supplementary Concept] OR (4-androsten-17-ol-3-one acetate) OR "testosterone-3-oxime" [Supplementary Concept] OR "testosterone isobutyrate" [Supplementary Concept] OR (Perandren M) OR (axeron)

#3 (**randomized controlled trial [pt] OR controlled clinical trial [pt] OR randomized controlled trials [mh] OR random allocation [mh] OR double-blind method [mh] OR single-blind method [mh] OR clinical trial [pt] OR clinical trials [mh] OR ("clinical trial" [tw]) OR ((singl* [tw] OR doubl* [tw] OR trebl* [tw] OR tripl* [tw]) AND (mask* [tw] OR blind* [tw])) OR ( placebos [mh] OR placebo* [tw] OR random* [tw] OR research design [mh:noexp] OR comparative study [mh] OR evaluation studies [mh] OR follow-up studies [mh] OR prospective studies [mh] OR control* [tw] OR prospectiv* [tw] OR volunteer* [tw]) NOT (animals [mh] NOT humans [mh])**

#1 AND #2 AND #3
